# *“I really wanted to be able to contribute something”*: understanding health science student motivations to create meaningful global health experiences

**Published:** 2012-09-30

**Authors:** Erin Hetherington, Jennifer Hatfield

**Affiliations:** Global Health and International Partnerships, University of Calgary, Canada

## Abstract

**Background:**

Global health is an area of increasing interest among health professionals, students and educators. This study aims to explore students’ motivations and experiences with an undergraduate global health research program in low and middle-income countries and to assess student learning and areas for program improvement.

**Methods:**

All students participating in the Global Health Research Program at the University of Calgary in the summer of 2009 were asked to participate in the study (n=11). In-depth interviews were conducted with students prior to departure and upon their return. Discourse analysis was used to identify interpretive repertoires and to determine how the use of repertoires improves our understanding of students’ experiences.

**Results:**

Prior to departure, students were highly motivated to “give back” to host communities. Upon return, students felt that their experience had been more about “building relationships” with others than individual contributions to hosts.

**Discussion:**

Students’ altruistic motivations dominated the discourse, and most students incorporated core concepts from a preparation course only after their international experience. Extensive preparation, supervision and follow-up support can mitigate many of the risks of short-term global health experiences while providing a safe opportunity for significant learning.

## Introduction

Global health is an area of increasing interest among health professionals, students and educators. Global health research focuses on transnational health issues requiring multidisciplinary cooperative responses and strives to promote health equity within and between populations.^1^ There has been a recent surge in demand for education in global health and international experiences for students, especially those wanting to travel to low and middle-income countries.^2^–^6^ These global health experiences are generally portrayed in a positive light and are viewed as providing increased professional development, cross-cultural training, personal growth, and increased appreciation for health inequities and preventative medicine, especially when the student is immersed in resource-limited settings.^2^,^6^–^11^ Several recent studies, however, have also highlighted a range of ethical concerns that emerge from such programs, including replicating colonial power structures, providing inappropriate interventions due to lack of proper engagement and assessment of community needs, draining host institutions limited resources, among other issues.^7^,^12^–^17^

As the number of global health programs increases, there is a growing call for a comprehensive curriculum to prepare students for their placements, and more structured supervision and evaluation of programs.^3^,^6^,^13^ There is currently wide variation in the preparation received by students.^3^,^18^–^22^ Reviews of global health competencies have revealed that no clear consensus exists among medical school curricula, and most recent articles outlining proposed competencies focus primarily on medical student electives.^3^,^20^,^23^

Moreover, evaluations of these programs can be difficult. Previous reviews of the effectiveness of international health programs found the majority of evaluations to be primarily descriptive, or based entirely on anecdotal self-reports or simple post-trip questionnaires that may miss the nuances of students’ understanding and learning.^6^,^9^,^10^,^14^,^24^,^25^ And although most studies report a high degree of enthusiasm for international programs, more research is needed to understand what motivates students to participate and how to best design and evaluate global health training programs for undergraduate students.

This paper describes a global health research program for undergraduate health science students at the University of Calgary. The program uses a three-phase approach which includes extensive preparation, supervision and follow-up. Discourse analysis of in-depth interviews both before and after the placement was used to examine how students view global health research, and how these perceptions change over the course of their experience. Understanding what motivates students to participate in global health experiences and the struggles they encounter can help shape policies regarding global health programs.

### Program description

The Global Health Program at the University of Calgary is founded on the notion that long-term relationships with international partners are the best environment for students to gain international experience. These equitable partnerships ensure that all research is based in local community needs and enhances the quality of the experience for both students and partners.^15^,^26^ This model stands in contrast to programs where students simply “drop-in” for cross-cultural exposure.^27^ This program encourages continuity, not only with international partners, but also with students. Each year a few students who participated in previous field placements are selected to return with new students. This ensures continuity for international partners, and also develops leadership skills in the returning students, who often play a mentoring role for the new students.

The program has three main components, a preparation course, an international placement for research, and a dissemination phase. The preparation course teaches core competencies for global health research in the following areas: global health inequities, capacity building, partnerships, knowledge translation, ethics and cultural competency. This curriculum was developed based on emerging literature and the work of the Canadian Coalition for Global Health Research.^15^,^18^,^26^–^28^ The preparation course is taught by both faculty and graduate students involved in global health research. During the preparation course, students develop research projects that are part of on-going programs of research in the host country.

The second phase is a three to four week international field placement in health care centres or university settings in Tanzania, Ethiopia or the Dominican Republic. Each group of students is accompanied by at least two staff members of the Global Health Program who oversee research projects, provide mentorship, and ensure the safety of the students as well as the overall success of the field experience. All research projects are developed based on the needs of our international partners, and include elements of capacity building and knowledge translation. Previous research projects have included an evaluation of the introduction of Rapid Diagnostic Tests for Malaria in a remote hospital in Tanzania, investigating cultural beliefs and practices related to HIV transmission among Maasai pastoralists, and examining the efficacy of child nutrition projects in the Dominican Republic.

After returning from their international placement, students must participate in the third phase, the dissemination phase, where they analyse the results of their work, and present the findings at academic conferences and/or publish their results.^29^,^30^ The preparation of all manuscripts, posters or abstracts is done in collaboration with host country researchers, who are listed as co-authors. Research results are also disseminated back to the host partner institution and used to inform future research needs. Approximately fifteen students participate every year, and each receives full funding for their trip.

## Methods

### Sample and interviews

All health science students participating in the summer global health research program in 2009 were asked for consent to be interviewed. Only one was unable to participate due to travel conflicts, leaving eleven participants. There were four male and seven female students, three in their 2^nd^ year of studies, seven in their 3^rd^, and one in their 4^th^ year. All students participated in two one-on-one, semi-structured interviews, one prior to departure and the second upon their return. Based on a review of the literature and the authors’ own experiences, the interview key was developed to explore the following themes: students’ professional and personal motivations, expectations and experiences; cross-cultural research; future plans and concepts of global health research.^6^,^8^,^10^,^24^,^31^ Due to the personal nature of the questions, and because the students knew each other very well, one-on-one interviews were chosen over a focus group approach. Focus groups are generally not recommended among groups with an established working relationship as they can emphasize existing group dynamics.^32^ Each 30–45 minute interview was conducted, recorded, and transcribed by the first author. Due to the small number of participants in the study, demographic information of participants is not linked to individual quotes to protect anonymity; students are identified by number only. The research protocol received ethics approval from the University of Calgary Conjoint Health Research Ethics Board.

### Analysis

Discourse analysis was used to examine how students discussed their expectations and experiences in global health. This method analyses the use of words beyond their literal meaning in order to examine how language helps construct and constrain identities, relationships and beliefs.^33^ For this study, Wetherell and Potter’s methods for identifying interpretive repertoires were used.^34^ An interpretive repertoire is an interconnected set of terms and descriptions organized around a central idea or metaphor.^34^,^35^ Interpretive repertoires are constructed from existing discourses about a topic but used in flexible ways depending on function and context.^36^ For example, global health research can be referred to as a set of activities done “for” a vulnerable community and other times as a set of activities done “with” a vulnerable community.

Participants’ responses were coded using broad themes and identifying commonly used terms, key metaphors and figures of speech. These were then analysed to identify patterns of similarity and variation at the individual level and across participants. The variation in how participants discussed specific topics formed the basis for developing the interpretive repertoires. Thematic coding and initial development of repertoires was done by the first author and checked for consistency and coherence by the second author. All data were coded using NVivo software (version 8; QSR International Pty Ltd).

This methodology was chosen because it allows an in-depth understanding of how students’ views of global health research are constructed and the consequences of these views.^35^ Specifically, this type of analysis shows how participants spoke about global health research, and the way in which they speak about global health research helps us understand their views about the topic, and more generally, the way they position themselves within their worldview of global health research. Interview keys were identical in the pre and post interviews allowing for comparison of students’ views before and after leading to insights into the impact of participation in the program.

## Results

### Identification of interpretive repertoires

Three main interpretive repertoires were identified regarding global health research, each of which focused on a central idea: global health research as “*Giving Back”*, global health research as “*Building Relationships”* and international travel as “*Self-centred Tourism”*. Each of these interpretive repertoires and their key attributes are presented in Table 1. All students used ideas and terms from each of these repertoires; however, some were more frequently used before they travelled and others only emerged after their international experience. For example, the “*Self-centred Tourism”* repertoire was dominant before travel, while “*Building Relationships”* emerged almost entirely after students returned. The *“Giving Back”* repertoire was equally present before and after travel. Because the pre and post-interview keys were virtually identical, the shift in the use of interpretive repertoires before and after travel is particularly salient.

#### Interpretive Repertoire #1: “Giving Back”

The central idea in this repertoire was a strong motivation to “give back” by sharing their expertise with the host community. Students were potentially drawing on the existing discourse around “giving back” commonly used in the media to highlight the charitable nature of a person or group, for example: American Idol “Gives Back”. Participants referenced organizations such as Médecins Sans Frontières (MSF or Doctors without Borders) and Dignitas International as models of good global health practice In this repertoire, the global health researcher was portrayed as an expert who can bring their knowledge and resources to the community:

I’d love to be able to do something to give back to the hospital that they would be able to use. [this research] would be really useful to the clinical officers for making a diagnosis, so is there a risk group and if I could find that out, and give something like that to the people at the hospital that they would actually use, that would be so wonderful. [Student 7 – pre-interview]

Global health research was portrayed as “real research”, in contrast to literature reviews and using data sets and spread sheets. Because of the connection to the community, global health research was seen as more worthy and likely to have a greater impact than large-scale analysis of health statistics. Global health research was described as “culturally sensitive”, which was characterized as a respect for the host community. In this repertoire, cultural sensitivity involved respecting the other culture, being open-minded and not judging cultural differences (Table 1).

This repertoire draws on notions of individual contribution, with the global health researcher being idealized to a certain extent as someone who enters the research setting and helps others in a respectful manner.

#### Interpretive Repertoire #2: “Building Relationships”

This repertoire focuses more on partnerships and shared learning than the individual desire to “give back”. Here, participants drew on core concepts present in the global health literature and presented in their preparation course. The emphasis was on being part of a team as opposed to individual contributions. In addition, participants spoke of building relationships with community members and working in partnership with others:

*I do actually feel that I made relationships that I normally wouldn’t have made before. (…) I think that watching people like [the graduate students] and how close they were with the staff, and how much they enjoyed it and the staff enjoyed it, and how much it benefitted their research and benefitted their research participants as well, I think that’s probably why I did it*.

[Student 1- post-interview]

Global health research was portrayed as an imperfect process with complications and setbacks that required a team effort. Building on the idea of global health research as a collaborative effort as opposed to an individual one, the perception of cross-cultural interactions is also different. In contrast to the more individualistic “culturally sensitive” view of respecting someone else’s culture seen in the “giving back” repertoire, the “building relationships” repertoire included the notion that experiencing another culture was an exchange of ideas that might help build understanding of one’s own culture. This included the realization that despite the fact that there might be elements of the other culture with which they were uncomfortable, such as racism or sexism, they still could and needed to work with those others (Table 1).

#### Interpretive Repertoire #3: “Self-centred Tourism”

Participants also referenced negative views of tourism for the third interpretive repertoire. Tourists were consistently painted as a different kind of traveller, and one who was not as altruistic as the global health researcher:

*I’m going there not as a tourist in my mind. I’m going there to work, and to do something specific, so I have a goal, so I’m very driven in that regard. I’m going there not with my family or friends, [but with] people I work with, colleagues and it’s Africa, (…), it’s a completely different environment, (…) this isn’t a resort, it’s a research compound (…), so a completely different feel I think. And just the perspective I’m going there with is completely different. I’m not going there on a vacation, I’m going there to learn and see new things and to work, so I think that, I think it will be a totally different experience*.

[Student 6 – pre-interview]

Tourism was viewed as selfish travel, whereas the travel the students were involved with had a purpose, and that purpose was to help others.

Tourists were often portrayed as not caring about the people who lived in the places they visited and were just in it to see the sights. Students strongly distanced themselves from tourists, noting that their travel was much more worthy. None of the students explicitly mentioned the fact that their participation in this field placement might be seen as tourism, or have some similar effects to tourism, whereas some of the students expressed discomfort with their own level of privilege compared to the people in their host country.

### Changes before and after the trip

The use of the three repertoires changed before and after the students’ trip. In some cases, students spoke about global health research in similar ways before and after. In other cases, there was a marked change in how students spoke about specific topics. At a specific level, before the trip, many students idealized global health research as “real” research that was more worthy than research involving statistics and literature reviews. However, upon return, the majority of students had changed this language to portray global health research as complex and “messy” (Table 1). On the repertoire level, the “building relationships” repertoire emerges almost entirely after the students’ trip (Table 1). However, it is not all students whose views have changed. In Table 2, two students are contrasted in their before and after answers to the question “What are your goals?/Did you accomplish them?” Student 1’s statements reflect the overall change in the group from the desire to contribute individually to the host community, to understanding that being part of a team may have a greater impact. Student 2, on the other hand, is focused on individual impact before and after her/his trip.

Among some students, there was a change or broadening of their views of cross-cultural interactions. Prior to departure, the emphasis was on being respectful, open-minded and non-judgemental of other cultures. This non-judgemental attitude is related to the notion of the global health researcher as an outsider who is “giving back” to a population different from their own. Many students retained this attitude upon their return. However, some students felt that they had learned more about their own culture by “building relationships” with people in another culture. In addition, some students recognized that even though they had the intention of respecting a different culture, they encountered elements of that culture with which they were uncomfortable, and they had to find strategies of working within elements they disagreed with. This is a more nuanced understanding of cross-cultural work that goes beyond an arms’ length non-judgemental approach, to one that sees and recognizes cultural differences and complexity of working within a system you may not agree with (Table 3).

Finally, many students specifically brought up things they had learned during the field placement. This included the importance of community engagement and strategies for understanding working with other cultures.

I think that the (…) thing I learned is that community engagement is important in research. Um, seeing some of the tensions that there were with [other external researchers working in the community], and seeing how the [other researchers] did some of their research and how the community was NOT happy about that research. And especially that the community said the [other researchers] were there in December that had been using their facilities, and not contributing anything. Like I was shocked at that. [Student 1 post-interview]

I think one thing that I came away with is a feeling of...you sort of learn more about your own culture when you experience a different culture. And you learn how to deal with different cultures better when you’re out of your own than when you’re in. When you’re the person who’s different, when you’re the one out of your element, you sort of appreciate how frightening it must feel, something that you can bring back when dealing with people of different cultures in Canada. [Student 7 - post interview]

## Discussion

The way in which participants draw on the three repertoires illustrates how they are positioning themselves in relation to their view of global health research. This is useful for educators and supervisors to better understand motivations, but also to help identify potential shortcomings or challenges students may face given this particular understanding of global health research.

### Shifts from an individual to collective experience

The variation in how students spoke about global health research is clearly indicated in the three repertoires. And although many elements of the repertoires were present both before and after travel, there were some overall changes that emerged as well. For example, the “building relationships” repertoire emerged almost entirely after travel. This repertoire is based primarily on sharing experiences and knowledge, which is in contrast to the “giving back” repertoire, which is primarily an individual view. “Giving back” focuses on how the individual can contribute to the greater good in a respectful way. Cross-cultural interactions are seen as being “non-judgemental” of the “other” culture, whereas in the “building relationships” repertoire, cross-cultural interactions are about sharing and learning about one’s own culture while learning about others. Research goes from being something that can be done to be “beneficial to the community”, to learning about different approaches to a problem and sharing expertise. Overall, this shift in repertoires signals a change from the individual contribution to a focus on a collective experience, shared not only within the group, but with the host community.

### Core concepts from preparation course integrated after travel

The preparation course introduces students to core concepts such as capacity building, partnerships, and knowledge translation. However, although students used these terms before travelling, they had a somewhat superficial understanding of them. Students expressed the desire to pursue research that was relevant to the community before they left, but only when they returned did they discuss engaging with the community in order to truly understand community needs and producing relevant research. So although the preparation course gave the students an understanding of the need for research to be relevant to the community, it was only the actual experience of meeting with community members that enabled students to understand the complexities of, and need for, community engagement in making research meaningful.

The preparation course also includes coaching on working cross-culturally. And although the course does not explicitly cover ideas such as cultural relativism or ethnocentrism, students are taught the importance of being respectful and open-minded. Before travel, almost all students spoke about being “non-judgemental” towards other cultures but only after travel did some of them realize that working effectively and respectfully within another culture might not necessarily mean agreeing with all elements of that culture. Also, it was only upon return that students were able to name strategies they used to work effectively across cultures both abroad and at home. In addition, most students showed a more comprehensive and sophisticated understanding of working cross-culturally when they attributed their learning about their own culture to working with others from a different culture.

Overall, students views shifted from a more idealized view of global health research before their trip, reflected in the “giving back” repertoire, to a more realistic view afterwards reflected by the “building relationships” repertoire”. This reflects a more nuanced understanding of global health research which emerged after students had experienced the challenges of carrying out their research in the field.

### Potential challenges with altruistic motivations

Altruism remains the dominant motivation for participation in this global health research program, which is consistent with other studies.^7^,^14^,^17^ And although students’ motivation to use their knowledge to help their host communities is commendable, it can also be unrealistic. A small undergraduate research project is unlikely to drastically affect the health or health care of a host population, and could potentially drain already limited resources. Previous research notes that the drain on the host’s resources is rarely evaluated and short-term research projects are often not rooted in community needs.^6^,^14^,^15^ This could lead to students being tempted to practice beyond their ability, or overestimate their ability to contribute, a concern raised by previous authors regarding international medical electives.^7^,^12^,^13^,^17^ Even though students were modest about their personal contributions, none of them reflected on the possibility that their presence might add to the workloads of the already busy local staff. And although this program provides substantial on-the-ground supervision and is designed to minimize demands on local institutions, there is no question that hosting a group of foreign researchers and students takes time and energy. Providing adequate supervision during an international experience is critical to ensuring that staff in low and middle-income settings are not further burdened by hosting international students.

Altruistic motivations can also lead to students feeling disempowered by their experiences. Many students were overwhelmed by the poverty they witnessed on their trip. And their altruistic motivations, in some ways, may have meant they were unprepared for the impotence they felt when actually confronted by poverty on this scale. Students’ focus on “giving back” and distancing themselves from tourists, can present them with internal conflicts. Many students used the “self-centred tourist” repertoire as a way of defining what they were **not**. And although none of the students in this study explicitly mentioned that they might have unintentionally been tourists in some ways, many were distinctly uncomfortable with their affluent position relative to that of the people in their host communities. A study by Petrosoniak et al. found that medical students participating in international health electives expressed similar discomfort with the possibility that they were practicing “medical tourism”.^7^ Managing expectations and the challenges students face when their expectations are not met is critical before, during and after any international experience. And although this was not anticipated, participating in the post-interview for this research acted as a kind of debrief for students when they returned home. It allowed a space for students to discuss their experiences from the trip, and reflect on any difficulties they had during the trip or during re-entry into their home culture.

### Conclusion

Our analyses highlight the need for educators to be aware of students’ views of themselves, their motivations to participate in international experiences in low and middle-income countries, and the difficulties that may arise from these perceptions. Taking these issues into consideration can help educators to minimize potential negative effects for students and meet their responsibilities towards sustainable global health education. The need for comprehensive preparation, supervision and debrief of international student experiences is not new.^3^,^4^,^11^,^19^ What our analyses reveal is an understanding of what motivates students to participate in global health experiences and the struggles they encounter. Understanding these factors can help shape policies regarding design of global health programs. Thorough preparation, supervision and post-trip support are essential elements to any successful experience.

Comprehensive preparation for students must go beyond logistics and safety considerations. Inequities in global health, capacity building, knowledge translation, ethics and cross-cultural training are key concepts for students to understand and integrate in order to be properly prepared for a successful and ethical international experience. The preparation students receive in this program provides a platform for the more nuanced learning they gain during their international experience. It would be impossible for any pre-departure course to eliminate culture shock or ensure a completely smooth experience. The point of preparation is to equip students with the proper knowledge and tools to handle the challenges they encounter, and learn from them.

On-going supervision can help ensure that research and other activities are carried out in an ethical manner and that students do not become a burden to hosts. In addition, it is important to consider that students may often be motivated by a strong desire to “give back”, but need adequate and on-going supervision to ensure they are not pressured or tempted to step beyond their ability and risk inflicting more harm than good. Supervisors provide essential oversight and ensure student safety, but can also help students reflect on their own limitations while in the field. Post-trip support and research dissemination are also key elements of any successful program. In order to sustain long-term partnerships with international collaborators it is critical to disseminate research results back to the host community or institution. Finally, as a result of this research, a formal debriefing component was added to our program. The debrief adds to the dissemination phase of the program by adding a space for students to reflect on their personal growth.

This research has provided insight into further program development internally, but can also serve to guide other educators who offer global health experiences to students. Understanding students’ motivations can highlight areas for preparation and potential program improvement. Preparation, supervision and follow-up support can help mitigate many of the risks of short-term global health experiences while providing a safe opportunity for significant and sophisticated learning.

## Figures and Tables

**Table 1:** Interpretive repertoires of students before and after the international experience (*n* = 11)

CENTRAL IDEA								
Global Health Research is “Giving Back”			Global Health Research is “Building Relationships”			Travel as “Self-centred Tourism”		

Key Terms	Pre	Post	Key Terms	Pre	Post	Key Terms	Pre	Post
* GH researchers give back to the population	8	10	* GHR means being part of a team that can give back	0	8	* Tourists go to see sights	6	2
* GHR is relevant to the community	7	7	* GHR means building relationships and partnerships with the community	0	9	* Tourists do not contribute to society	9	0
* GHR is *real* research (vs. statistics and lit reviews)	5	1	* GHR is complex	2	8			
			* GHR involves learning from others	1	6			
*Cultural sensitivity is being respectful and non-judgemental	7	10	* Cultural awareness					
			* Discomfort with elements of other cultures (racism, sexism)	0	6			
			* Learning about one’s own culture	7	10			

**Table 2:** Students 1 and 2 on motivations and ability to accomplish them

Pre	Post
Q: *What are your goals?*	Q: *Were you able to accomplish your goals?*

**Student 1** *I really wanted to, I didn’t want to go and just be a tourist, I wanted to go and contribute something and understand what was going on, and not to, not to be a deadweight Delores, I really wanted to able to, uh, contribute something.*	*I think I showed the hospital staff how committed we are to doing this project and how much we enjoyed their involvement and appreciated their involvement, so I’m not sure that I contributed to the actual findings or the development of research, but maybe being part of the team, and being friendly with them and trying to tell them what we were using their research for.*
Repertoire: Global Health Research is “giving back”	Repertoire: Global Health Research is “building relationships”

**Student 2** *Health care is a human right and I think that (…) what can I do within the world to be okay with myself, and okay with what I can contribute and (…) I do what I do because I’m pursuing justice and I do what I do because I want to invoke positive change in the world.*	*The hardest thing for me this trip was, actually the last night in the country when we went out and were in [the city] and having a couple of drinks and seeing this little child with her mother, I mean she must have been one, one and a half, and this was the reality of this kid in the middle of the night near bars, and I could do nothing. Like I couldn’t do anything to help this kid, and it was this disempowering moment where I felt that the next time that I’m in a position like this, I’ll be able to do something like this.*
Repertoire: Global Health Research is “giving back”	Repertoire: Global Health Research is “giving back”

**Table 3:** Student 4’s views of cross-cultural work

Pre	Post
Q: *What is the biggest challenge of working cross-culturally?*	

*Biggest challenge… um… probably just, avoiding being ethno-centric, maybe, I guess. (…) we’ll be asking the parents questions about child rearing and so often, for the past data, parents will say that they hit their children with metal rods and sticks and things, and I just have to remember that it’s a different place and you can’t see them as being a bad parent, just because of that.*	Well (…) racism in *where we were+ was pretty huge and um... (…) I think it’s like a very... Canadian thing to not... I don’t know... pick out race I guess. (…) I would ask (…) do you think *that people in your country] are racist, and they would say, oh of course not, we’re not racist at all, but then they’d say things, like, educated people, people you wouldn’t expect to say.
Repertoire: Global Health Research is “giving back”	Repertoire: Global Health Research is “building relationships”
